# Particulate Matter is Not Associated With Disease Worsening in Patients With Multiple Sclerosis: Retrospective Results From a Clinic‐Based Cohort in the Northeastern United States

**DOI:** 10.1002/brb3.71878

**Published:** 2026-11-10

**Authors:** Sophie Lebiedz, Brian Healy, Bonnie Glanz, Tanuja Chitnis, Evan Madill

**Affiliations:** ^1^ Brigham MS Center Brigham and Women's Hospital Boston Massachusetts USA; ^2^ Biostatistics Center Massachusetts General Hospital Boston Massachusetts USA; ^3^ Harvard Medical School Boston Massachusetts USA; ^4^ Multiple Sclerosis Center University of Washington Seattle Washington USA

**Keywords:** multiple sclerosis, neurodegenerative, particulate matter, pollution

## Abstract

**Objective:**

Exposure to air pollution is linked to relapses in multiple sclerosis (MS). We hypothesized that greater exposure to fine particulate matter (PM2.5) is associated with a faster time to progression independent of relapse activity (PIRA) and the time to an expanded disability status scale (EDSS) score of 6 or greater in persons with MS (pwMS).

**Methods:**

Adult patients who lived in the northeastern United States and were seen at the Brigham MS Center in Boston, MA, USA were included. Monthly PM2.5 levels were identified for every zip code in the northeastern United States between 2017 and 2023 using an open‐source satellite‐derived dataset. The relationship between average annual PM2.5 exposure and PIRA and the time to an EDSS score of 6 or greater was modeled using Cox proportional hazards regression.

**Results:**

The cohort included 3040 pwMS with an average age of 49.5 and a median EDSS of 2.7. Average baseline PM2.5 exposure was 5.8 µg/m^3^. Increasing levels of PM2.5 exposure in pwMS were not associated with time to PIRA using either baseline PM2.5 levels (unadjusted hazard ratio (HR): 1.00 (95% CI: 0.95, 1.06); *p* = 0.875) or time‐varying PM2.5 levels (unadjusted HR: 0.98 (95% CI: 0.93, 1.03); *p* = 0.390). After adjusting for age, sex, disease duration, EDSS, and number of relapses in the previous year, the results remained consistent for baseline PM2.5 levels (HR: 1.01 (95% CI: 0.96, 1.06); *p* = 0.704) and time‐varying PM2.5 levels (HR: 0.98 (95% CI: 0.93, 1.04); *p* = 0.539). There was no association between time to EDSS of 6 or greater and baseline level (HR: 1.02 (95% CI: 0.93, 1.11; *p* = 0.730) or time‐varying PM2.5 level (HR: 1.01 (95% CI: 0.92, 1.10; *p* = 0.854). After adjusting, results remained similar for both baseline (HR: 1.03 (95% CI: 0.94, 1.13); *p* = 0.499) and time‐varying PM2.5 level (HR: 1.01 (95% CI: 0.92, 1.10); *p* = 0.870).

**Conclusion:**

No relationship between higher PM2.5 exposure and the time to PIRA or EDSS 6 or greater in a large cohort of pwMS living in the northeastern United States was identified.

## Graphical abstract

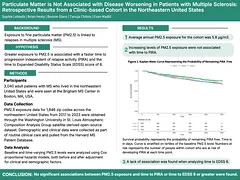

## 1 Introduction

Air pollution is defined as a mixture of exogenous liquids, gases, and solid particles present in both indoor and outdoor air. This includes particulate matter (PM), ground‐level ozone, nitrogen oxides, carbon monoxide, and sulfur oxides. PM is classified by size; coarse particulates (PM10) are less than 10 µm in diameter, fine particulates (PM2.5) are less than 2.5 µm, and ultrafine particulates (PM0.1) are less than 1 nm in diameter (Sierra‐Vargas and Teran 2012). PM2.5, specifically, has been studied for its role in increased oxidative stress and inflammation in the central nervous system; higher levels have been found to be correlated with the development of neurodegenerative diseases such as Alzheimer's disease and Parkinson's disease (Calderón‐Garcidueñas et al. 2008; Krzyzanowski et al. 2024). This may be related to the ability of PM2.5 to cross the blood‐brain barrier to drive microglial activation (Mühlfeld et al. 2008).

Persons with MS (pwMS) accrue disability through two main mechanisms: relapse‐associated worsening (RAW) and progression independent of relapse activity (PIRA). Recent evidence has shown that PIRA is the primary cause of disability worsening in MS, even among those with relapsing remitting disease (RRMS) (Lublin et al. 2022). Notably, PIRA is associated with microglial activation, creating a potential mechanism by which PM2.5 exposure might worsen progression in pwMS (Singhal et al. 2025).

Several groups have examined the potential correlation between air pollution exposure and MS relapse. In 2003, Oikonen et al. were among the first to report a link, suggesting that PM10 exposure increases the susceptibility to infections and, subsequently, relapse occurrence (Oikonen et al. 2003). Later, a study conducted in Lombardy, Italy, observed an increase in relapse‐related hospitalizations among RRMS patients in the days following high PM10 exposure (Angelici et al. 2016). Roux et al. (2017) reported a positive correlation between PM10 levels and MS relapse occurrence in Strasbourg, France (Roux et al. 2017). Januel et al. (2021) observed similar results in Paris, especially in patients under 30 years of age (Januel et al. 2021).

Despite these efforts, no studies to date have investigated the relationship between chronic air pollution exposure in pwMS and PIRA or EDSS score increase. The objective of this study was to evaluate whether higher levels of PM2.5 exposure were associated with disability worsening in MS. To address this question, we investigated the association between PM2.5 exposure and the time to PIRA, as well as the time to an EDSS score of 6 or greater in a large cohort from the northeastern United States.

## 2 Methods

### 2.1 Research Ethics and Informed Consent

This study was approved by the Mass General Brigham Institutional Review Board under a secondary use protocol (2025P001218). Data were collected as part of routine clinical care and stored in the Harvard MS Patient Database (2002P001045).

### 2.2 Participants

Patients aged 18 or older seen at the Brigham MS Center (Boston, MA, USA) who were diagnosed with MS or clinically isolated syndrome (CIS) by their treating MS physician were considered for inclusion. Patients were required to have a clinic visit between 2017 and 2020 and have a home address in New England. New England is a six‐state region in the northeastern United States comprising Connecticut, Maine, Massachusetts, New Hampshire, Rhode Island, and Vermont. Patients were excluded if they did not have at least one follow‐up visit in‐person at the Brigham MS Center.

### 2.3 PM2.5 Exposure Estimation

PM2.5 exposure data for all 1846 zip codes across the northeastern United States from 2017 to 2023 was obtained through the Washington University in Saint Louis Atmospheric Composition Analysis Group satellite‐derived open‐source dataset and the corresponding R package from The MITRE Corporation (Bedford, MA) (Van Donkelaar et al. 2021; Liberatore. et al. 2022). Patients lived in 595 unique zip codes. Patient zip codes at baseline clinic visit were matched to the corresponding zip code‐level pollution data. The pollution level was available for each month, and each measurement of pollution level was included in the analysis.

### 2.4 Outcomes and Clinical Data Source

The primary outcome measure was time to PIRA. PIRA was defined as an increase in expanded disability status scale (EDSS) of 1.5 for those with a baseline of 0, 1.0 for those with EDSS between 1.0 and 5.0, or a 0.5 increase for those with baseline EDSS of 5.5 or greater in the absence of a documented relapse (Müller et al. 2025). EDSS worsening had to be sustained for at least 365 days. Further, there could not have been relapses in the 30 days before any of the confirmation visits. The EDSS score was re‐baselined at any PIRA event, RAW event, or any sustained improvement in EDSS over at least 180 days.

The secondary outcome of this study was the first visit with an EDSS score of 6 or greater. Only subjects with an EDSS less than 6 at baseline were included in the analysis.

Demographic and clinical data were pulled from the Harvard MS Patient Database; this includes clinical measures, such as EDSS, MS subtype, and relapses, which were recorded at each clinic visit.

## 3 Statistical Analysis

A Cox proportional hazards model was used to estimate the association between the PM2.5 level and time to PIRA for all patients. Patients who did not have PIRA were censored at the earliest time to last clinic visit or January 1, 2024, since this was the end of the pollution data. The PM2.5 level was included in the analysis in two ways: the pollution measurement immediately after the baseline clinic visit was included in a model as a cross‐sectional predictor, and the monthly pollution measurements were included as a time‐varying predictor. Unadjusted hazard ratios and hazard ratios adjusted for age, sex, disease duration, baseline EDSS, and number of relapses in the previous year were estimated. Hazard ratios above 1 indicate that the hazard of PIRA increased with increasing PM2.5 levels. The same models were refit to estimate the association between PM2.5 level and time to first visit with EDSS of 6 or greater among patients with EDSS less than 6 at baseline. Statistical significance was defined as an alpha level of 0.05, and all tests were run in R version 4.4.2 (www.r‐project.org).

## 4 Results

Characteristics for the 3040 patients included in this study are presented in Table 1. Most patients had RRMS (73.1%), and the mean (SD) age was 49.4 (12.6), with a relatively long mean (SD) disease duration (15.1(11.3)). Most patients were white (88.2%) and female (74.0%), reflecting the overall population at the Brigham MS Center. Average disability was modest, with a mean (SD) EDSS of 2.7 (2.2).

**TABLE 1:** Demographic characteristics of subjects at first available visit with pollutant information.

N	3040
Age (mean (SD))	49.4 (12.6)
Sex (N (%))	
Female	2250 (74.0)
Male	788 (25.9)
Other	2 (0.1)
Race	
American Indian or Alaska Native	5 (0.2)
Asian	164 (0.5)
Black or African American	186 (6.1)
East Asian or South‐East	6 (0.2)
More than one race	66 (2.2)
Native Hawaiian or other Pacific Islander	3 (0.1)
South Asian	10 (0.3)
White	2680 (88.2)
Unknown or not reported	70 (2.3)
Ethnicity	
Hispanic	148 (4.9)
Not Hispanic	2804 (92.2)
Unknown	88 (2.9)
Disease duration (mean (SD))	15.1 (11.3)
EDSS (mean (SD))	2.7 (2.2)
Disease category	
CIS	129 (4.2)
PPMS	158 (5.2)
PRMS	22 (0.7)
RRMS	2223 (73.1)
SPMS	508 (16.7)
PM2.5 (mean (SD))	5.8 (1.5)

Abbreviations: CIS = clinically isolated syndrome, EDSS = expanded disability status scale, PM2.5 = particulate matter ≤ 2.5 µm, PPMS = primary progressive multiple sclerosis, PRMS = primary relapsing multiple sclerosis, RRMS = relapsing remitting multiple sclerosis, SD = standard deviation, SPMS = secondary progressive multiple sclerosis.

Average annual PM2.5 exposure for the cohort was 5.8 µg/m^3^. PM2.5 concentration ranged from 2.6 to 10.6 µg/m^3^.

The time to PIRA among our sample is shown in Figure 1. The time to PIRA among our sample is shown in Figure 1. Patients were grouped based on the tertiles of the baseline PM2.5 level. Increasing levels of PM2.5 exposure in pwMS were not associated with time to PIRA using either baseline PM2.5 level (unadjusted hazard ratio (HR): 1.00 (95% CI: 0.95, 1.06); *p* = 0.875) or time‐varying PM2.5 level (unadjusted hazard ratio (HR): 0.98 (95% CI: 0.93, 1.03); *p* = 0.390). After adjusting for age, sex, disease duration, EDSS, and number of relapses in the previous year, the results remained consistent for both the baseline level (adjusted HR for baseline level: 1.01 (95% CI: 0.96, 1.06); *p* = 0.704) and time‐varying PM2.5 level (adjusted HR for time‐varying level: 0.98 (95% CI: 0.93, 1.04); *p* = 0.539). A similar lack of association was found when analyzing time to EDSS 6 for the 2,484 patients with EDSS less than 6 at their baseline visit. In unadjusted analysis, there was no association between time to EDSS 6 and either baseline PM2.5 level (Unadjusted HR: 1.02 (95% CI: 0.93, 1.11; *p* = 0.730) or time‐varying PM2.5 level (Unadjusted HR: 1.01 (95% CI: 0.92, 1.10; *p* = 0.854). After adjusting for age, sex, disease duration, EDSS, and number of relapses, the association with time to EDSS 6 remained similar for baseline PM2.5 level (adjusted HR: 1.03 (95% CI: 0.94, 1.13; *p* = 0.499)) and time‐varying PM2.5 level (adjusted HR: 1.01 (95% CI: 0.92, 1.10; *p* = 0.870)).

**FIGURE 1:**
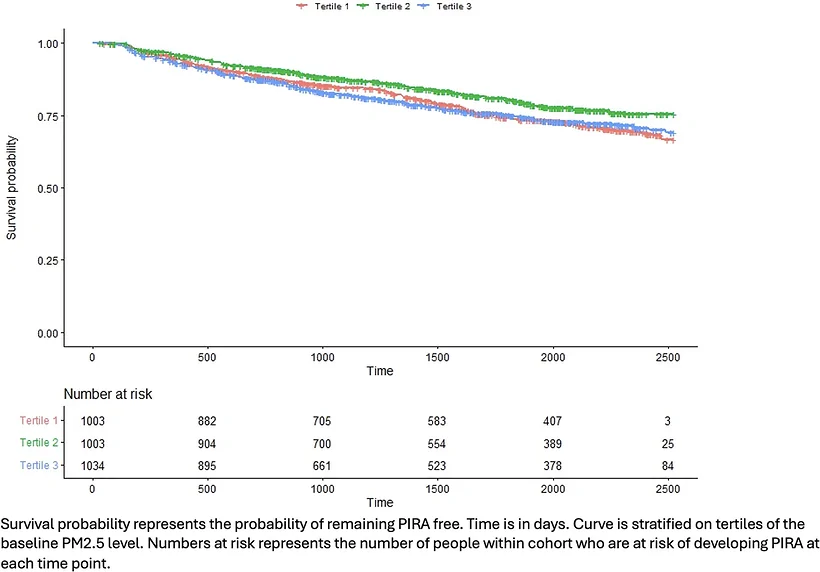
Kaplan‐Meier curve representing the probability of remaining PIRA free.

## 5 Discussion

This study investigated the potential association between PM2.5 exposure and progressive disease activity in MS in patients living in the northeastern United States. No correlations between PM2.5 exposure and time to PIRA or time to EDSS 6 or greater were found.

There are several potential explanations for the lack of correlations found in our study. First, the average annual PM2.5 exposure in our cohort was relatively low at 5.8 µg/m^3^. The U.S. Environmental Protection Agency limit is 9.0 µg/m^3^, and, between 2010 and 2020, the average concentration across the United States was 8.7 µg/m^3^ (U.S. Environmental Protection Agency 2016). Therefore, there may not have been sufficient heterogeneity or pollutant exposure in the cohort to find an underlying association, particularly if there is a threshold effect. Additionally, the duration of follow up for many patients may have been too short to detect a small effect on PIRA or EDSS score, which often develop over many years. Another possible explanation is that there is no relationship between PM2.5 exposure and MS disease progression, despite being observed in other neurological conditions.

Several limitations of this study should be considered. This study did not account for potential confounders that could be associated with PIRA and unevenly distributed across counties, such as smoking status and socioeconomic status, though these unmeasured confounders would likely lead to a stronger association rather than a weaker one (Belbasis et al. 2015). Another limitation is the granularity of the PM2.5 data. Since wildfire smoke is likely fairly homogeneous at the county‐level and PM2.5 exposure from motor vehicle traffic is heterogeneous even at 1 km^2^, we may have under‐ or over‐estimated industrial pollution at the zip code level. This means that the average concentration across a zip code may not accurately represent the exposure experienced by individual patients. Also, personal exposure can vary widely based on factors such as local pollution patterns, time spent outdoors, and proximity to major stationary sources of PM emissions (Börü et al. 2020). Finally, patient mobility was not taken into consideration. Although only a small fraction of subjects changed zip code over the course of the study (127/3040), there might be some exposure misclassification for the participants that moved.

An important strength of this study is the large sample size and its ability to identify PIRA at scale in a real‐world cohort of pwMS. Despite the biological plausibility, no evidence of an association between PM2.5 exposure and PIRA or faster time to EDSS 6 or greater was found. Further study is warranted, particularly globally, and potentially in regions of the United States where PM2.5 levels exceed the recommended threshold. Nevertheless, it is notable that PM2.5 concentration is not clearly linked to disease progression at what are considered safe levels for fine particulate matter.

## Author Contributions

**Sophie Lebiedz**: conceptualization, data curation, methodology, investigation, writing – original draft preparation, writing – review and editing. **Brian Healy**: formal analysis, methodology, resources, software, writing – review and editing. **Bonnie Glanz**: project administration, writing – review and editing. **Tanuja Chitnis**: conceptualization, writing – review and editing. **Evan Madill**: supervision, conceptualization, methodology, writing – review and editing.

## Funding

The authors have nothing to report.

## Conflicts of Interest

T.C. has received compensation for consulting from Biogen, Novartis Pharmaceuticals, Roche Genentech and Sanofi Genzyme. She has received research support from the National Institutes of Health, National MS Society, US Department of Defense, Novartis Pharmaceuticals, Octave Bioscience, Roche Genentech, Sanofi and Tiziana Life Sciences. All other authors declare no conflicts of interest.

## Data Availability

The data that support the findings of this study are available from the corresponding author, Brian Healy, upon reasonable request at bchealy@mgh.harvard.edu.
